# A Cross-Sectional Observational Study of Gingival Color Patterns in the South Indian Population

**DOI:** 10.7759/cureus.67340

**Published:** 2024-08-20

**Authors:** Hamilton Arokia Raj, Ponsekar Abraham Anandapandian, Ranjani Thillaigovindan, Sai Chaitanya Raj B.

**Affiliations:** 1 Prosthodontics, Thai Moogambigai Dental College and Hospital, Chennai, IND

**Keywords:** pink aesthetics., gingival color, dual shade guide, color threshold, cielab color space

## Abstract

Background: Gingival aesthetics or pink aesthetics requires a prosthodontic approach to ensure an appealing smile with an optimal muco-gingival appearance by the use of colored materials with gingival shades to match adjacent soft tissues. However, the selection of this adhesive gingival-colored material becomes complex owing to the wide range of gingival color guides and shade tabs currently available on the market.

Aim: The study aims to assess the variation in gingival color between two specific regions on the anterior gingival surface through the use of a digital color assessment method. Furthermore, the study seeks to investigate the potential requirements for an innovative soft tissue dual shade guide system.

Methodology: Fifteen participants were examined with an external light source set up in a 45-degree optical configuration. The Frontal view intraoral photographs were taken with a digital Canon 70D camera using a cheek retractor. The photo was white balanced using the color sorter tool in the software (Adobe Photoshop CS6®), and the second quadrant was cropped, two regions were selected (free gingival margin and marginal gingiva) and used for all samples for standardization. The color data were represented in terms of L*, a*, and b* coordinate axes values following the CIELAB color system. The recorded color coordinates were then examined using SPSS software, version 24 (IBM Corp., Armonk, NY).

Results: The mean and standard deviation of the coordinate axes were as follows: for L1, 52.33 ± 12.92; for a1, 30.06 ± 4.81; for b1, 18.00 ± 3.89; for L2, 44.53 ± 11.01; for a2, 36.13 ± 7.92; and for b2, 18.26 ± 6.70. Statistically significant differences were found between the L*, a*, and b* color coordinates with a color difference (ΔE) beyond the clinical acceptance (ΔE > 3.7) threshold of ΔE = 4.88, mainly for a* values.

Conclusions: Within the limitations of this study, significant color differences were observed between the selected regions. The a* coordinate was found to be statistically significant (+6.07), indicating a shift towards a lighter shade of redness (+a) in the color-opponent dimensions of redness-greenness within the CIELAB color space system.

## Introduction

Esthetic concerns in fixed or removal prostheses and implant-supported restorations have focused predominantly on the anterior region largely influenced by facial appearance, physical features of the artificial tooth, and the fabrication process [1]. The health of the soft tissues surrounding the prosthetic restorations is equally important as the aesthetics, including the shape, contour, and color of the prosthetic teeth. The characterization of the soft tissue, gingiva in particular mimicking the natural appearance demands an accurate selection of color and communication precisely with the lab experts to achieve good esthetic outcomes [2-4].

Gingival aesthetics or pink aesthetics requires a prosthodontic approach to ensure an appealing smile with optimal mucogingival characterization using colored materials to match adjacent soft tissues [3,4]. Several methods have been established over the years to determine the color and shade of tooth and gingival complex, including visual dental shade guide, dental shade tabs, spectrophotometry, and digital color analysis. Visual dental shade guides either as customized or prefabricated ones are most commonly used for tooth color or gingival color determination that represent the full spectrum of colors naturally occurring in the human dentition [4-6].

Previous studies have shown human eye detects the color difference (3.7 △E unit) intra-orally under standardized observation conditions. These Visual methods are subjective and depend on age, color blindness, eye fatigue, extreme light conditions, the intensity of the light source, angulation of incidence light, and several physical variabilities [3,4,7,8]. Hence, the selection of gingival-colored material becomes complex owing to the wide range of color guides and tabs currently available on the market. Nonetheless, the use of color standards for soft tissues at the craniofacial region was not fully recognized due to several factors such as varying hue and Chroma, size, the thickness of the soft tissue at specific areas, ethnic background, and individualization of different color characteristics [1,4,5,8].

Digital cameras have demonstrated potential as an alternative to traditional visual color evaluation by offering precise shade calibration and minimizing human error. By utilizing the CIE L*a*b* color system, developed by the International Commission on Illumination (Commission Internationale de l’Éclairage), this digital method provides a baseline value for color matching. In the CIELAB color space, colors are quantified numerically with L* representing lightness, a* indicating the green (−) to red (+) axis, and b* denoting the blue (−) to yellow (+) axis. This approach enhances the correlation with visual assessments and ensures accurate color quantification [1,3,9].

Recent research on the assessment of human gingiva color has indicated that the CIELAB color system may offer better reliability compared to the Munsell three-color system. However, it is noted that the exact instrumental CIE L*a*b* values have not yet been identified due to the wider range of gingival color compared to tooth color [9, 10]. Furthermore, there is currently no published illustrative range of soft tissue color shades, although it has been suggested to combine shades during processing to better replicate natural color. As a result, the present study aimed to assess the difference in gingival color between two specific areas in the anterior gingival region using digital color assessment methods and to consider the potential need for a novel dual soft tissue shade guide system.

## Materials and methods

Study participants

**Figure 1:**
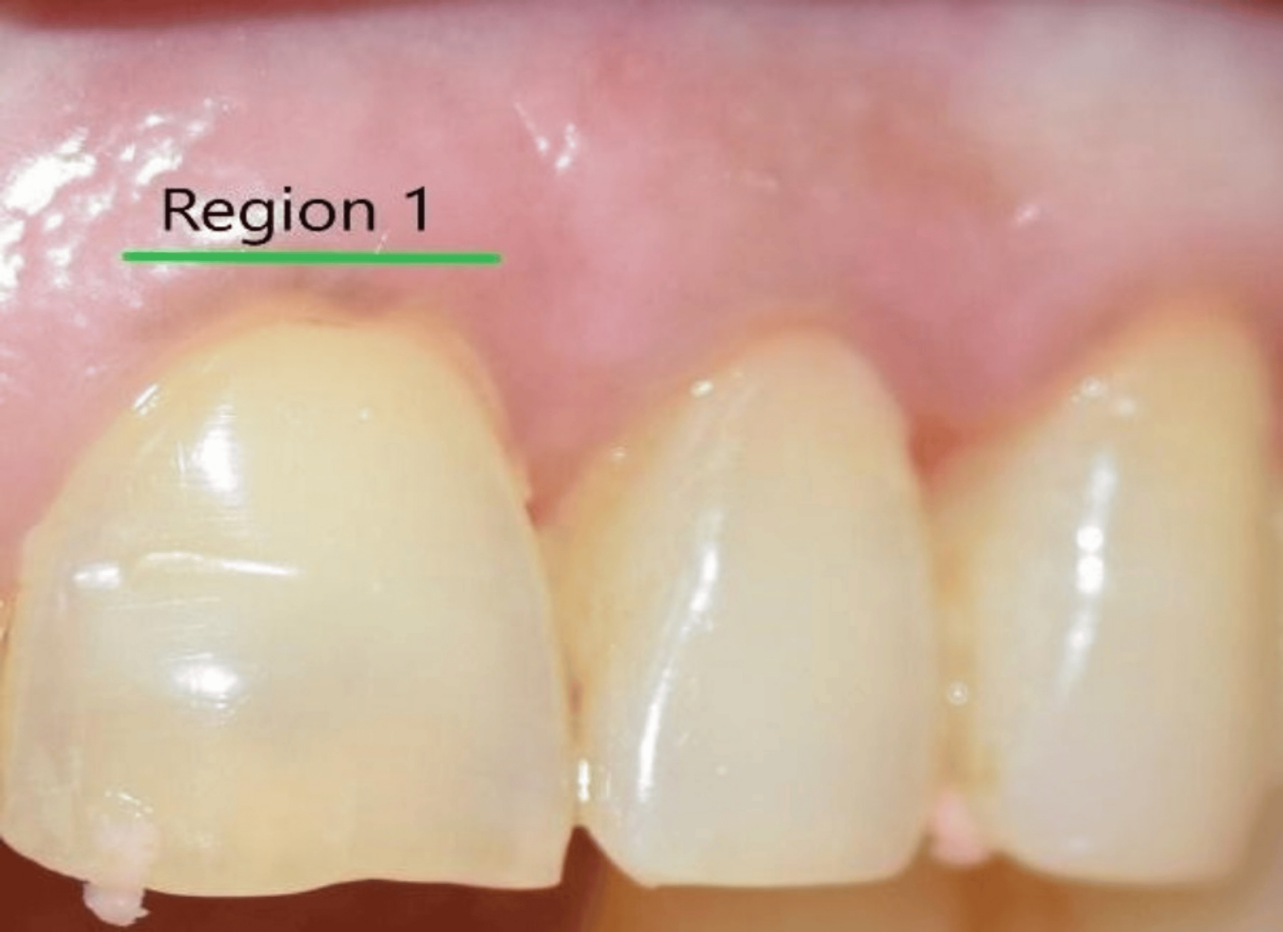
Image showing Region 1 used for digital analysis using the CIELAB color shade system. CIELAB, Commission Internationale de l‘Eclairage

**Figure 2:**
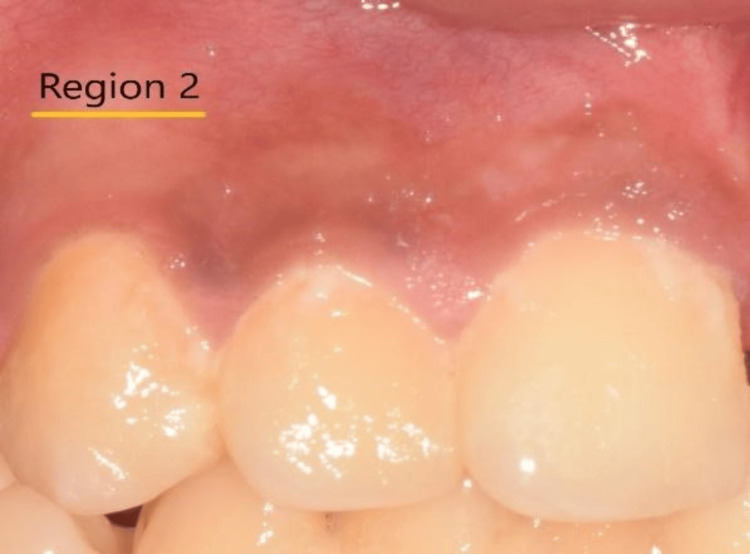
Image showing Region 2 used for digital analysis using the CIELAB color shade system. CIELAB, Commission Internationale de l‘Eclairage

Fifteen Participants were examined in a dental chair with an external light source set up in a 45-degree optical configuration for visual assessment (Sample size was calculated using g Power analysis). The intraoral photographs were taken with a Canon 70D camera to measure the color of the gingiva between the free gingival margin (Region 1, Figure 1) and marginal gingiva (Region 2, Figure 2) over the maxillary anterior tooth region. The study received ethical approval from the Institutional Review Board of Thai Mookambigai Dental College (approval No. 228/2022/IEC/TMDCH, November 24, 2022) and was conducted over three months.

The frontal view image was captured using a cheek retractor to enhance the visibility of the gingiva while the surface of the mucosa was air-dried and cleaned to avoid reflection from saliva on the surface.

All participants with the absence of any relevant systemic diseases, no visible melanin pigmentation at the specific region, no restored or endodontic treated teeth, no gingival recessions or any oral Mucocutaneous lesions, and those who brush twice a day regularly with full mouth dental plaque index equal to or less than 25%, and a gingival or bleeding index of less than 10% were included in the study. Participants with a known history of smoking, tobacco chewing, with visible signs of gingival inflammation, pregnant women, alterations in alveolar or keratinized mucosa (amalgam tattoo, marked melanin pigmentation), and patients using medications that could alter oral soft tissue were excluded from the present study.

The color data were expressed in terms of L*, a*, and b* values by the CIELAB color space. To ensure a consistent white balance, a known-value gray card was utilized. The photo underwent white balancing in Adobe Photoshop, and the second quadrant was cropped in the region of the attached gingiva 2.5 mm apical to the crest of the marginal gingiva, close to the specified region, and this was used for all samples to maintain standardization. The L*, a*, and b* values based on the Commission Internationale de l‘Eclairage (CIELAB) and the color differences (DE) were measured and used for subsequent analyses.

Statistical analysis

The data captured from the color coordinates was subjected to analysis using SPSS software, version 24.0 (IBM Corp., Armonk, NY). Descriptive analysis was utilized to determine the mean, standard deviation, and range of the color coordinates. A Kolmogorov-Smirnov analysis was employed to evaluate the normal distribution of the data. Additionally, the Pearson coefficient was utilized to assess the correlation between specific regions, with statistical significance set at a *P*-value of less than 0.05.

## Results

**Figure 3:**
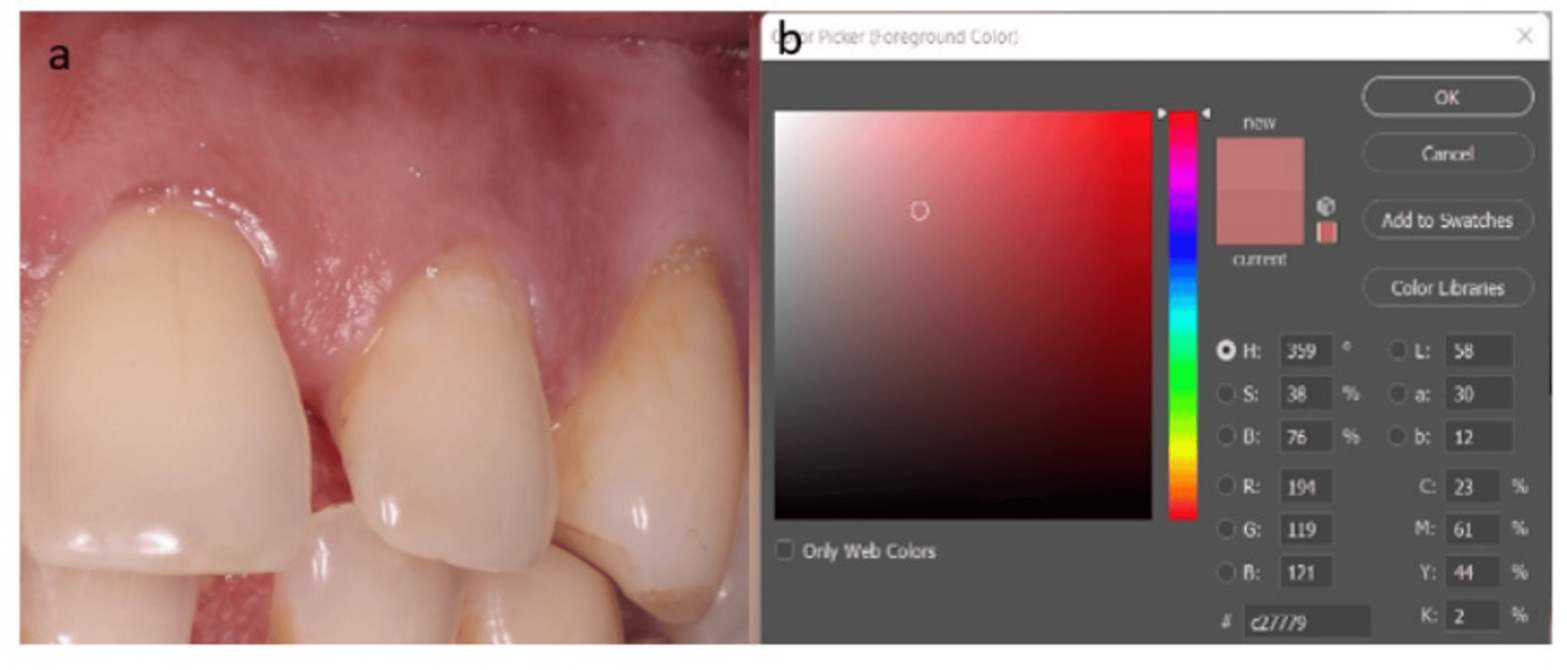
Region 1 (a) - L*a*b* analysis coordinate axes scores using the CIELAB color shade system (b). CIELAB, Commission Internationale de l‘Eclairage

The statistical values for the measured gingiva CIELAB values are provided in Table 1, including the mean, standard deviation, maximum, and minimum values. The gingival color was examined at two specified regions (L1a1b1, L2a2b2) and was bounded by the following maximum and minimum coordinates: L1 - minimum 33.0 and maximum 74.0; a1 - minimum 22.0 and maximum 37.0; and b1 - minimum 10.0 and maximum 25.0. The mean ± standard deviation for L1 was 52.33 ± 12.92, for a1 was 30.06 ± 4.81, and for b1 was 18.00 ± 3.89 (Figure 3) and for L2: minimum 27.0 and maximum 62.0; a2: minimum 20.0 and maximum 47.0; b2: minimum 5.0 and maximum 29.0.

**Table 1:** Color values of measured gingival color at two different regions (n = 15). Kolmogorov-Smirnov test. ^*^P < 0.05 (Significant).

Color data	Mean	Maximum value	Minimum value	Standard deviation	*P*-value
L1	52.33	74.00	33.00	12.92	0.034
a1	30.06	37.00	22.00	4.81	
b1	18.00	25.00	10.00	3.89	
L2	44.53	62.00	27.00	11.01	
a2	36.13	47.00	20.00	7.92	
b2	18.26	29.00	5.00	6.70	
L*	48.430	68.000	30.000	11.965	
a*	33.095	42.000	21.000	6.365	
b*	18.130	27.000	7.500	5.295	

**Figure 4:**
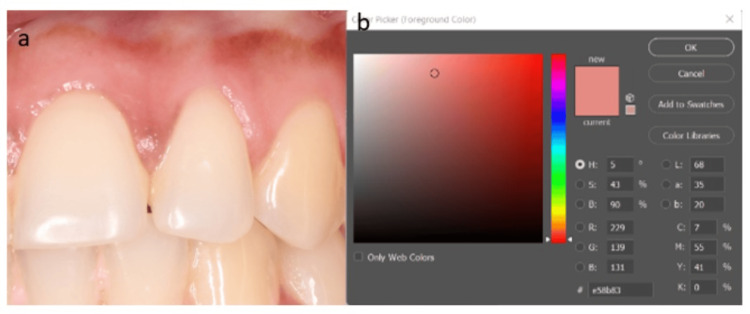
Region 2 (a) - L*a*b* analysis coordinate axes scores using the CIELAB color shade system (b). CIELAB, Commission Internationale de l‘Eclairage

The mean ± standard deviation values were 44.53 ± 11.01 for L2, 36.13 ± 7.92 for a2, and 18.26 ± 6.70 for b2 (Figure 4).

The coordinate axes are significant at *P *< 0.05 in the Kolmogorov-Smirnov test, thus confirming the normality in the distribution of the data (*P*-value).

The gingival color values at the two specified regions were measured using the CIELAB values and the DE value between the groups was calculated. For L*, the mean coordinate was 48.43 ± 11.97; for a*, it was 33.10 ± 6.37; and for b*, it was 18.13 ± 5.30. However, differences in the a* coordinate were statistically significant, with a value of 6.07 at *P* < 0.05. Additionally, the present study found a ΔE value of 4.88 units, indicating a clear mismatch in color measurement (Table 2).

**Table 2:** Mean CIELAB color coordinates and differences. DE values were found to be 4.88 units. Formula used: DE = [(L*)2 + (a*)2 + (b*)2]1/2. DE, △E; CIELAB, Commission Internationale de l‘Eclairage

Samples	Coordinate	Square values	DE values
L* (L2-L1)	-7.80	60.84	4.887625
a* (a2-a1)	6.07*	36.8449	
b* (b2-b1)	0.26	0.0676	

**Figure 5:**
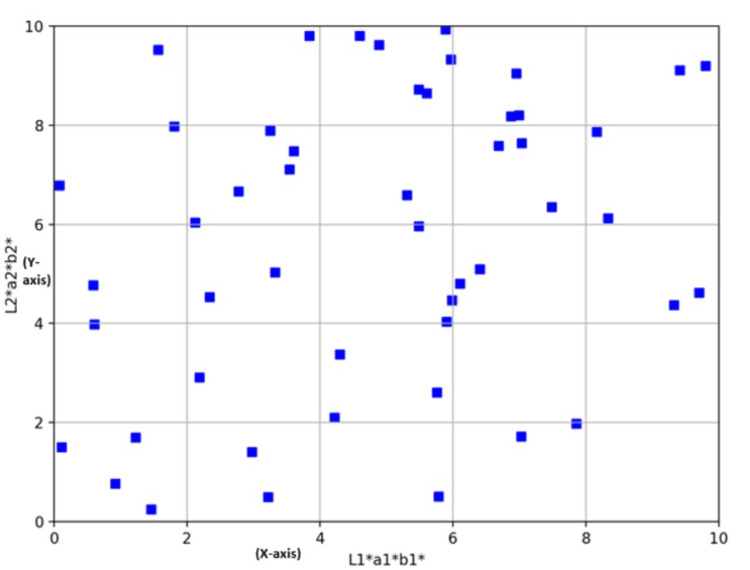
Graph showing the coordinate axes points of specific regions.

The Pearson coefficient calculator between two specific regions revealed a correlation coefficient (R) of 0.6787, with a *P*-value of 000037. This result was considered statistically significant at the commonly accepted *P*-value threshold of 0.05. The analysis indicated a moderate positive correlation, suggesting that higher X variable scores (L1*a1*b1*) were associated with higher Y variable scores (L2*a2*b2*) and X variable scores (L1*a1*b1*) and Y variable scores (L2*a2*b2*) (Figure 5).

## Discussion

The gingival color determination made by visual observation methods over the years lacked reliability, validity, reproducibility, and accuracy. Color assessment using the photometric method, digital cameras, and spectrophotometric measurements showed potential as an alternative method to visual color evaluation by providing accurate shade calibration and reducing human error. The combination of the digital method with the CIE L*a*b* color system for color matching was preferred over spectrometric measurement despite limitations such as illumination control, film processing, and other parameters to ensure numerical color quantification by establishing better correlation with visual standards [1,3,9].

Polo et al. [1], Huang et al. [3], Cardoso et al. [7], Naranjo et al. [9], and Ho et al. [11] examined the color distribution of healthy human gingiva and found that, in comparison to the attached gingiva region 2.5 mm apical to the crest of the marginal gingiva used in the current study, the midpoint of the keratinized gingiva had a slightly lower amplitude of all the CIELAB color coordinates (L*, a*, b*). Three color coordinates are used in the CIELAB system: b* for the blue/yellow axis, a* for the green/red axis, and L* for brightness. These coordinate axes agree with prior research despite differences in earlier techniques for measuring gingival color [3,7,8,9].

Polo et al. [1], Huang et al. [3], Ito et al. [12], and Sailer et al. [13] proposed several gingival guide systems based on Lab* coordinates obtained using a spectrophotometer. Their reported ΔE* thresholds ranged from 5.8 to 19.5 units. The present study's ΔE value of 4.88 units indicates a significant color mismatch during color measurement, which, while less than the thresholds reported in previous studies, may still be imperceptible to the human eye when aided by digital photographs. Visual comparative approaches have been used clinically to select gingival color, however, lack validity and reliability. Cardoso et al. [7], Naranjo et al. [9], Mahn et al. [14], Denissen et al. [15], and Perez et al. [16] observations were consistent with our study results that recommended the use of electronic devices or photography method with software guided CIELAB color shade system as an alternative for obtaining accurate L*a*b* variables for the gingival shade guide.

Studies by Cardoso et al. [7], Ghinea et al. [8], Bayindir et al. [10], Perez et al. [16], and Amer et al. [17] had shown significantly lower coverage errors similar to the present study that evaluated color differences within a series of digital photographs at specified regions on the anterior gingival surface that had been altered by Software (Adobe Photoshop). From these observations, it is evident that Digital imaging systems using definition cameras with standardized settings and the use of gray cards for white balance are becoming more popular in measuring the shade of teeth due to their reliability, accuracy, and reduced errors when combined with the proper standardization techniques though spectrophotometry has shown greatest validity and reliability for tooth or gingival color measurement.

Cardoso et al. [7], Anand et al. [18], and Hein et al. [19] showed significant variations beyond the expected coordinate axes in agreement with this study where a* coordinate difference in color is higher presenting more towards lighter shade (L-) of redness (+a) in the color-opponent dimensions of redness-greenness on the CIELAB Color space system while studies by Naranjo et al. [9], Ho et al. [11], and Calvo Box showed significant differences in the color coordinate b* between the study groups. This could be due to the influence of study population, site-specific region, analysis methodology, and gingival characteristics like biotype, melanin pigmentation, and amount of keratinization. 

The present study had limitations such as the measurement of coverage errors using CIEDE2000 (ΔE00), recommended in 2001 by the International Commission on Illumination (CIE), the photograph guiding principle, the position of the patients, smaller sample size, and several demographic factors such as age, gender, and ethnicity. Another limitation was the point of analysis which was 2.5 mm apical to the crest of marginal gingiva defined without any standard guides as a possible interference attributed to limited gingival shade guides. Nonetheless, this study showed the need for a dual shade guide system for gingival shade selection presenting more towards lighter shade of redness (a* coordinate axes) on the redness-greenness color space by the CIELAB color space system.

Strengths

This study exhibits several strengths that underscore its importance in prosthodontics. It introduces a novel approach by employing digital color assessment methods to evaluate gingival color variation, which could lead to the creation of more accurate gingival shade guides. Methodological rigor is demonstrated through the controlled environment used for capturing intraoral photographs, including the consistent use of a Canon 70D camera and a 45-degree optical setup. The application of white balance with a gray card and standardized cropping enhances data reliability. The study's use of the scientifically recognized CIE Lab* color system minimizes human error and variability compared to traditional visual methods. Objective data analysis, supported by statistical software, provides a robust evaluation of the color data. By focusing on clinical relevance, the study addresses critical aspects of prosthodontic practice, particularly in achieving optimal aesthetic outcomes. Furthermore, the findings emphasize the need for a dual shade guide system for gingival shade selection, filling a significant gap in current practice. This study's preliminary data lay a foundation for future research, potentially inspiring further advancements in this field.

Limitations

This study on gingival color patterns among the South Indian population has limitations, including a small sample size and a focus on a specific ethnic group, which limits generalizability. The use of digital photographs and software analysis introduces potential errors despite standardization efforts. Only two gingival regions were analyzed, excluding other potentially relevant areas. Challenges in ensuring consistent white balance and potential observer bias in image processing further affect the results. The study's exclusion criteria and lack of longitudinal data also limit applicability. Additionally, the proposed dual shade guide system requires further validation in clinical settings. Larger, more diverse studies using advanced measurement techniques are needed to improve reliability and applicability.

## Conclusions

In conclusion, the present study revealed substantial differences in color shade observed between the selected regions among which a* coordinates were found to be statistically significant (+6.07) presenting more towards lighter shade (L-) of redness (+a) in the color-opponent dimensions of redness-greenness on the CIELAB Color space system. This emphasized the need for further evaluation on a larger scale to establish L*a*b* color coordinates for constructing well-designed gingival color guides using digital methods, thus creating an aesthetically pleasing appearance.

This study enhances clinical practice by providing a precise method for assessing gingival color, crucial for achieving optimal aesthetic outcomes in dental restorations. The findings highlight the need for a dual shade guide system, enabling more accurate color matching and reducing the likelihood of mismatched prosthetics. For patients, this leads to natural-looking restorations, boosting confidence and satisfaction. Additionally, the study's objective approach streamlines the color-matching process, resulting in more efficient treatments and fewer adjustments. Overall, it improves both the aesthetic and functional outcomes of dental restorations, benefiting both clinicians and patients
